# Coenzyme Q10 status, glucose parameters, and antioxidative capacity in college athletes

**DOI:** 10.1186/s12970-020-0334-3

**Published:** 2020-01-10

**Authors:** Chien-Chang Ho, Ching-Yu Tseng, Hung-Wun Chen, Yi-Wen Chiu, Ming-Chih Tsai, Po-Sheng Chang, Ping-Ting Lin

**Affiliations:** 1 Department of Physical Education, Fu Jen Catholic University, New Taipei, 24205 Taiwan; 2 Research and Development Center for Physical Education, Health and Information Technology, College of Education, Fu Jen Catholic University, New Taipei, 24205 Taiwan; 3 Department of Nutrition, Chung Shan Medical University, Taichung, 40201 Taiwan; 4 Graduate Program in Nutrition, Chung Shan Medical University, Taichung, 40201 Taiwan; 5 Department of Nutrition, Chung Shan Medical University Hospital, Taichung, 40201 Taiwan

**Keywords:** Athletes, Coenzyme Q10, Glucose parameters, Antioxidant capacity, Sport nutrition

## Abstract

**Background:**

Glycemia is related to energy production during exercise. Coenzyme Q10 is an antioxidant that participates in adenosine triphosphate synthesis in mitochondria. The aim of this study was to investigate the level of coenzyme Q10, glucose parameters, and antioxidant capacity in athletes.

**Methods:**

This study was designed as a cross-sectional study. Well-trained college athletes (*n* = 43) and age-gender matched healthy subjects (*n* = 25) were recruited from a college. The levels of glucose parameters, oxidative stress, antioxidant enzymes activity, Trolox equivalent antioxidant capacity (TAC), and coenzyme Q10 status were measured in the present study.

**Results:**

The athletes had a significantly lower level of white blood cells (WBC) coenzyme Q10 than the healthy subjects (0.34 ± 0.24 vs. 0.65 ± 0.43 nmol/g, *p* < 0.01); however, no significant difference was detected in plasma coenzyme Q10 between the two groups. Regarding the glucose parameters, the athletes had significantly higher values for HbA1c (5.5 ± 0.3 vs. 5.3 ± 0.3%, *p* < 0.05) and quantitative insulin sensitivity check index (QUICKI, 0.37 ± 0.03 vs. 0.34 ± 0.03, *p* < 0.05), and lower homeostatic model assessment-insulin resistance (HOMA-IR, 1.5 ± 0.8 vs. 2.9 ± 3.8, *p* < 0.05) than the healthy subjects. A higher level of TAC was found in the athletes (serum, 5.7 ± 0.3 vs. 5.4 ± 0.2 mM Trolox; erythrocyte, 10.5 ± 0.6 vs. 10.0 ± 0.5 mM Trolox, *p* < 0.05). In addition, WBC coenzyme Q10 status was significantly correlated with catalase activity (*r* = 0.56, *p* < 0.01), GPx activity (*r* = 0.56, *p* < 0.01), serum TAC (*r* = 0.54, *p* < 0.01), fasting glucose (*β* = − 1.10, *p* < 0.01), HbA1c (*β* = − 0.82, *p* < 0.01), HOMA-IR (*β* = − 1.81, *p* < 0.01), and QUICK (*β* = 0.08, *p* < 0.01).

**Conclusions:**

Athletes may suffer from a marginal coenzyme Q10 deficiency, and the level was related to glycemic control and antioxidant capacity. Further interventional studies are needed to clarify an adequate dose of coenzyme Q10 supplementation in athletes to optimize their coenzyme Q10 status and athletic performance or recovery during exercise.

## Background

Adequate nutrients and energy intake are required for the health and exercise performance of athletes [1]. Carbohydrates are the major fuel for athletes, and athletes with high insulin sensitivity may increase carbohydrate storage in the form of glycogen for athletic performance [2]. Blood glucose and insulin responses may be involved in the regulation of carbohydrate and lipid oxidation to produce energy for skeletal muscle contractions during exercise [3, 4]; therefore, glycemic control in athletes is an important factor of sports nutrition. In the human body, pancreatic β-cells are responsible for blood glucose regulation, but they contain lower level of antioxidative enzymes and are sensitive to oxidative damage [5]. Many studies have indicated that athletes have increased production of reactive oxygen species during high-intensity training, which contributes to high oxidative stress, thereby resulting in muscle protein loss, fatigue, injury, and reduced physical performance [6–8].

Coenzyme Q10 is a lipid-soluble nutrient that participates in the mitochondrial respiratory chain for adenosine triphosphate (ATP) synthesis [9, 10]. Athletes require immediate energy for exercise; however, few studies have investigated the coenzyme Q10 status of athletes. Our previous clinical study of individuals with type 2 diabetes found that the relationship of coenzyme Q10 concentration with glycemic regulation may be medicated through its antioxidant capacity [11]. Thus, the correlation between coenzyme Q10 status and glucose and antioxidant activity in athletes should be explored. The purpose of the present study was to investigate the level of coenzyme Q10, glucose parameters, and antioxidant capacity in athletes and examine the relationships among these factors. We hypothesized that the level of coenzyme Q10, glucose parameters, and antioxidant capacity in athletes might differ from those in healthy subjects.

## Methods

### Participants and study design

This study was designed as a cross-sectional study. Well-trained athletes and healthy college students were recruited from Fu Jen Catholic University in Taiwan. The inclusion criteria were as follows: athletes were required to train for more than 12 h every week, and healthy subjects were required to not train regularly (less than 12 h every week) and were age- and gender-matched with the athletes. The exclusion criteria were age younger than 18 years, the consumption of nutritional or coenzyme Q10 supplements, and the use of anti-hyperlipidemia or anti-thrombin agents. This study was approved by the Institutional Review Board of Fu Jen Catholic University, Taiwan (FJU-IRB C105132). Each subject participated in the study after providing written informed consent.

### Demographic assessments

A questionnaire was used to determine the characteristics, including gender, age, and lifestyle habits, of the subjects. The height, weight, and waist circumference of each subject were measured, and then, the body mass index and waist hip ratio were calculated. We used the International Physical Activity Questionnaires (IPAQ) to assess subjects’ activity. The unit used was metabolic equivalent of task (MET)-minutes/week. One MET was defined as the amount of oxygen consumed when sitting at rest and was equal to 3.5 ml oxygen/kg body weight/minutes [12].

### Hematologic measurements

Fasting blood samples were collected in vacutainers containing K2-EDTA anticoagulant (Becton Dickinson, Franklin Lakes, NJ, USA) or sodium fluoride (Sparsh Mediplus, Mumbai, Maharashtra, India); serum was separated in the tube without anticoagulant. Plasma, buffy coat layer, erythrocytes, and serum samples were obtained after centrifugation at 4 °C and 3000 rpm for 15 min. White blood cells (WBC) were obtained from buffy coats layers using red blood cells (RBC) lysis buffer [13].

Hematologic data, such as glucose, insulin, lipid profiles, albumin, blood urea nitrogen, creatinine, creatine phosphokinase, lactate dehydrogenase, alkaline phosphatase, glutamic oxaloacetic transaminase, glutamic pyruvic transaminase, and uric acid levels, were analyzed by an automated chemistry analyzer (Roche, Cobas 8000, Basel, Switzerland), glycated hemoglobin (HbA1c) was analyzed by an automated glycated hemoglobin analyzer (Trinity Biotech, Bray, Co., Wicklow, Ireland), and total lymphocyte count was measured by a hematology analyzer (Sysmex, XN-3000, Kobe, Japan). Then, we further calculated markers of insulin resistance, and the following formulas were used: homeostatic model assessment-insulin resistance (HOMA-IR) = glucose (mmol/L) × insulin (μU/mL)/22.5; homeostatic model assessment-β-cell function (HOMA-β) = 20 × insulin (μU/mL)/(glucose (mmol/L) – 3.5) [14]; and quantitative insulin sensitivity check index (QUICKI) = 1/[log insulin (μU/mL) + log glucose (mg/dL)] [15].

### Oxidative stress and antioxidant enzymes measurements

Malondialdehyde as an oxidative stress indicator, was determined in plasma and erythrocytes by the thiobarbituric acid reactive substance method [16]. Antioxidative enzymes activity was determined in erythrocytes by calculating changes in the absorbance value of the substance by spectrophotometry. The activities of superoxide dismutase (SOD), catalase (CAT), and glutathione peroxidase (GPx) were measured at 325 nm, 240 nm, and 340 nm, respectively [17–19]. A BCA protein assay kit (Thermo, Rockford, IL, USA) was used to determine the protein concentration in erythrocytes to adjust antioxidative enzyme activity. A Trolox equivalent antioxidant capacity assay was used to determine the total antioxidant capacity (TAC) in serum and erythrocytes at 730 nm [20].

### Coenzyme Q10 status measurement

Coenzyme Q10 status was measured by high-performance liquid chromatography (HPLC) with an ultraviolet detector. The plasma coenzyme Q10 analysis was performed according to Littarru et al. [21]. The WBC pellet sample was added to 100 μL propanol until it was homogeneous and was then measured using the same process as that used for plasma. The analysis column used was a LiChroCART®RP-18 (Merck, Germany), and the ultraviolet detector was set at 275 nm.

### Statistical analysis

This was an exploratory study. Descriptive statistics are presented as the mean ± standard deviation (median) or percentages. The Shapiro-Wilk test was used to examine the normality of data distribution. Student’s t-test or Mann-Whitney rank sum test was used to examine the differences in coenzyme Q10, glucose parameters, oxidative stress, and antioxidant capacity between the two groups. A chi-square test or Fisher’s exact test was used to compare the differences in categorical variables. Spearman’s rank order correlation analyses and simple linear regression analyses were used to examine the correlations between coenzyme Q10 status and glucose parameters or antioxidant capacity in athletes. All statistical tests in the study were conducted using SigmaPlot software (version 12.0, Systat, San Jose, California, USA). The statistical significance level was set at a *p* value ≤0.05.

## Results

### Subject characteristics

The characteristics of the athletes and healthy subjects are shown in Table 1. Forty-three athletes and twenty-five healthy subjects with a mean age of 20 years participated in this study. The ratio of males to females was approximately 2:1. The types of sports were taekwondo and soccer, accounting for 49 and 51%, respectively. There was no significant difference in anthropometric or lifestyle habit data between the two groups. With regard to the activity, the athletes had a significantly higher MET of total physical activity, moderate-intensity activity, and vigorous-intensity activity (*p* < 0.01) and a significantly lower time spent sitting than healthy subjects (*p* < 0.01). For hematological values, the levels of blood urea nitrogen and HDL-C were significantly higher in athletes than in the healthy group (*p* < 0.05); in contrast, the levels of alkaline phosphatase and triglyceride were significantly lower in athletes than those in the healthy group (*p* < 0.05).

**Table 1:** Characteristics of subjects

	Athletes	Healthy subjects
Male/Female (n)	27/16	17/8
Age (years)	19.7 ± 1.3 (20.0) ^1^	19.7 ± 1.1 (20.0)
Sports		
Taekwondo (n, %)	21 (49%)	–
Soccer (n, %)	22 (51%)	–
Anthropometry		
BMI (kg/m^2^)	22.1 ± 2.1 (21.5)	22.3 ± 2.6 (22.1)
Waist hip ratio	0.79 ± 0.04 (0.79)	0.79 ± 0.06 (0.78)
Activity (MET-minutes/week)		
Total physical activity	17,610.5 ± 7781.7 (16,800.0)*	7351.7 ± 5643.2 (6178.0)
Moderate-intensity activity	3384.3 ± 2390.5 (2880.0)*	1443.2 ± 1682.9 (800.0)
Vigorous-intensity activity	12,485.6 ± 5759.6 (12,480.0)*	4345.6 ± 3606.0 (3600.0)
Time spent sitting (min/day)	45.9 ± 34.8 (35.0)*	349.1 ± 237.8 (325.7)
Lifestyle habits		
Current smokers (n, %)	4 (9%)	6 (24%)
Current drinkers (n, %)	3 (7%)	3 (12%)
Hematology		
Albumin (g/dL)	49.9 ± 2.5 (50.0)	50.5 ± 3.2 (51.0)
Total lymphocyte count (cell/mm^3^)	2177.0 ± 638.8 (2052.8)	2229.9 ± 635.5 (2163.4)
BUN (mmol/L)	5.2 ± 1.0 (5.4)*	4.6 ± 0.9 (4.6)
Creatinine (μmol/L)	79.6 ± 17.7 (79.6)	79.6 ± 17.7 (79.6)
eGFR (mL/min/1.73 m^2^)	103.7 ± 13.5 (103.4)	102.0 ± 10.5 (104.2)
ALP (U/L)	68.4 ± 27.3 (61.0)*	81.1 ± 19.6 (81.0)
GOT (U/L)	19.0 ± 5.3 (18.0)	22.9 ± 9.5 (22.0)
GPT (U/L)	15.3 ± 7.6 (13.0)	20.2 ± 10.1 (21.0)
Uric acid (μmol/L)	350.9 ± 71.4 (356.9)	327.1 ± 77.3 (333.1)
TC (mmol/L)	4.4 ± 0.8 (4.2)	4.4 ± 0.8 (4.4)
TG (mmol/L)	0.74 ± 0.51 (0.62)*	0.89 ± 0.44 (0.710)
LDL-C (mmol/L)	2.5 ± 0.7 (2.3)	2.6 ± 0.7 (2.4)
HDL-C (mmol/L)	1.8 ± 0.4 (1.8)*	1.6 ± 0.3 (1.6)
CPK (U/L)	315.6 ± 239.5 (213.0)	398.0 ± 573.2 (267.0)
LDH (U/L)	200.3 ± 38.9 (193.0)	210.2 ± 45.9 (211.0)

^1^ mean ± SD (median). *p < 0.05. ALP, alkaline phosphatase; *BMI* body mass index; *BUN* blood urea nitrogen; *CPK* creatine phosphokinase; *eGFR* estimated glomerular filtration rate; *GOT* glutamic oxaloacetic transaminase; *GPT* glutamic pyruvic transaminase; *HDL-C* high density lipoprotein-cholesterol; *LDH* lactate dehydrogenase; *LDL-C* low density lipoprotein-cholesterol; *MET* metabolic equivalent; *TC* total cholesterol; *TG* triglyceride

### Glucose parameters, oxidative stress, and antioxidant capacity

The levels of glucose parameters, oxidative stress, antioxidative enzymes activity, and total antioxidant capacity are shown in Table 2. The athletes had significantly higher levels of HbA1_C_ (*p* = 0.01) and QUICKI (*p* < 0.01) than the healthy subjects, while significantly lower levels of insulin and HOMA-IR were found in the athletes (*p* < 0.01). Although there was no significant difference in oxidative stress and antioxidant enzymes activity between the two groups, the athletes had significantly higher levels of TAC in the serum and erythrocytes than the healthy subjects (*p* < 0.01).

**Table 2:** Glucose parameters, oxidative stress, and antioxidant capacity of the subjects

	Athletes	Healthy subjects
Glucose parameters		
Fasting glucose (mmol/L)	4.8 ± 0.4 (4.8)	5.1 ± 0.6 (4.9)
HbA1_C_ (%)	5.5 ± 0.3 (5.5)*	5.3 ± 0.3 (5.3)
Insulin (pmol/L)	47.9 ± 22.2 (41.0)*	88.2 ± 97.7 (66.7)
HOMA-IR	1.5 ± 0.8 (1.2)*	2.9 ± 3.8 (2.0)
HOMA-β (%)	106.6 ± 39.2 (98.6)	172.1 ± 136.4 (138.6)
QUICKI	0.37 ± 0.03 (0.37)*	0.34 ± 0.03 (0.35)
Oxidative stress		
Plasma MDA (μM)	2.4 ± 0.4 (2.4)	2.4 ± 0.4 (2.4)
Erythrocyte MDA (nmol/mg protein)	3.8 ± 0.3 (3.8)	3.8 ± 0.3 (3.8)
Antioxidant capacity		
SOD (U/mg protein)	28.6 ± 10.9 (27.9)	27.9 ± 9.4 (26.9)
CAT (U/mg protein)	36.2 ± 6.1 (35.6)	34.4 ± 7.1 (35.2)
GPx (U/mg protein)	22.7 ± 4.1 (22.8)	21.1 ± 2.8 (20.9)
Serum TAC (mM Trolox)	5.7 ± 0.3 (5.8)*	5.4 ± 0.2 (5.4)
Erythrocyte TAC (mM Trolox)	10.5 ± 0.6 (10.5)*	10.0 ± 0.5 (10.0)

^1^ mean ± SD (median). * *p* < 0.05. CAT, catalase; *GPx* glutathione peroxidase; *HbA1*_*C*_ glycated hemoglobin; *HOMA-IR* homeostatic model assessment-insulin resistance; *HOMA-β* homeostatic model assessment-β-cell function; *MDA* malondialdehyde; *QUICKI* quantitative insulin sensitivity check index; *SOD* superoxide dismutase; *TAC* total antioxidant capacity

### Coenzyme Q10 status

**Fig. 1:**
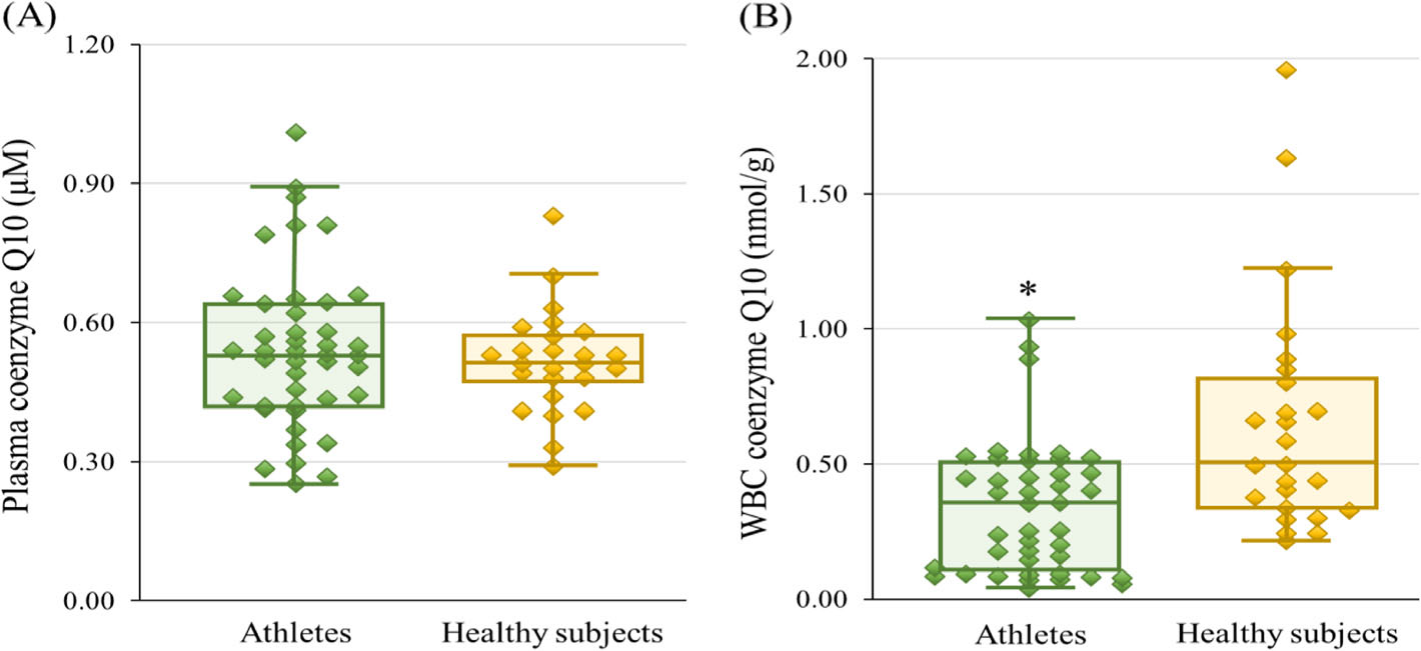
Coenzyme Q10 status (**a**) Plasma coenzyme Q10; (**b**) WBC coenzyme Q10. * *p* < 0.01. WBC, white blood cells

Figure 1 shows the coenzyme Q10 status in the athletes and healthy subjects. The athletes had significantly lower level of WBC coenzyme Q10 than the healthy subjects (0.34 ± 0.24 nmol/g vs. 0.65 ± 0.43 nmol/g, *p* < 0.01). However, there was no significant difference in plasma coenzyme Q10 (0.54 ± 0.17 μM vs. 0.52 ± 0.11 μM, *p* = 0.56).

### Correlations between coenzyme Q10 status and antioxidant capacity

The correlations between coenzyme Q10 status and antioxidant capacity in athletes are shown in Table 3. Coenzyme Q10 status was significantly positively correlated with CAT activity (plasma coenzyme Q10, *r* = 0.50, *p* < 0.01; WBC coenzyme Q10, *r* = 0.56, *p* < 0.01), GPx activity (plasma coenzyme Q10, *r* = 0.49, *p* < 0.01; WBC coenzyme Q10, *r* = 0.56, *p* < 0.01), and serum TAC (plasma coenzyme Q10, *r* = 0.37, *p* < 0.05; WBC coenzyme Q10, *r* = 0.54, *p* < 0.01).

**Table 3:** Correlations between coenzyme Q10 status and antioxidant capacity in the athletes

Antioxidant capacity	Coenzyme Q10 status	
	Plasma coenzyme Q10 (μM)	WBC coenzyme Q10 (nmol/g)
SOD (U/mg protein)	−0.03 ^1^	− 0.12
CAT (U/mg protein)	0.50**	0.56**
GPx (U/mg protein)	0.49**	0.56**
Serum TAC (mM Trolox)	0.37*	0.54**
Erythrocyte TAC (mM Trolox)	0.02	−0.07

^1^ *r*, Spearman rank order correlation coefficients. *p < 0.05; **p < 0.01

*CAT* catalase; *GPx* glutathione peroxidase; *SOD* superoxide dismutase; *TAC* total antioxidant capacity; *WBC* white blood cells

### Correlations between coenzyme Q10 status and glucose parameters

**Fig. 2:**
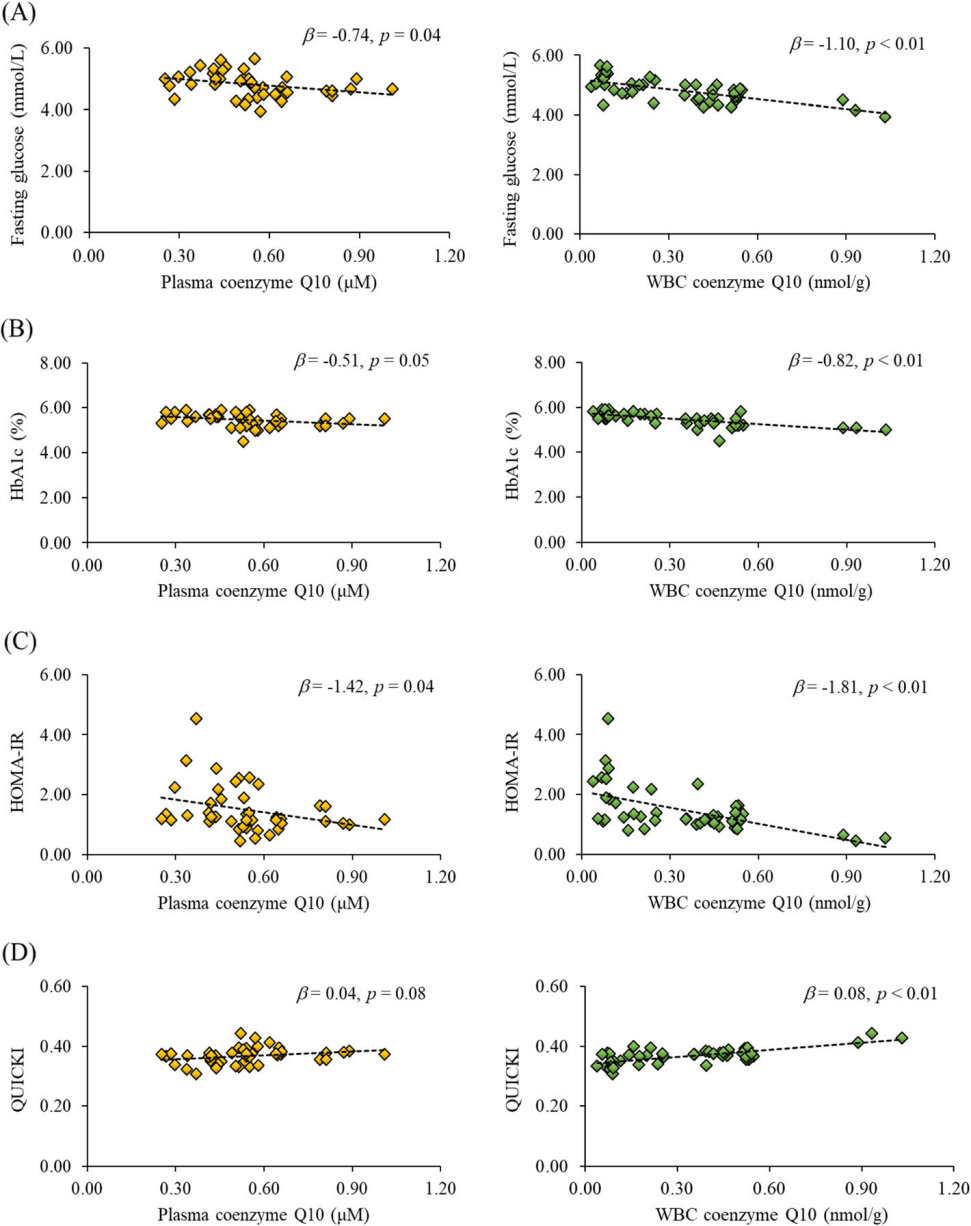
Correlations between coenzyme Q10 status and glucose parameters in the athletes. **a** Correlations between coenzyme Q10 status and fasting glucose. **b** Correlations between coenzyme Q10 status and HbA1_C_. **c** Correlations between coenzyme Q10 status and HOMA-IR. **d** Correlations between coenzyme Q10 status and QUICKI. HbA1_C_, glycated hemoglobin; HOMA-IR, homeostatic model assessment-insulin resistance; QUICKI, quantitative insulin sensitivity check index

The correlations between coenzyme Q10 status and glucose parameters in athletes are shown in Fig. 2. Coenzyme Q10 status was significantly negatively correlated with glucose parameters, such as fasting glucose (plasma coenzyme Q10, *β* = − 0.74, *p* = 0.04; WBC coenzyme Q10, *β* = − 1.10, *p* < 0.01), HbA1c (plasma coenzyme Q10, *β* = − 0.51, *p* = 0.05; WBC coenzyme Q10, *β* = − 0.82, *p* < 0.01), and HOMA-IR (plasma coenzyme Q10, *β* = − 1.42, *p* = 0.04; WBC coenzyme Q10, *β* = − 1.81, *p* < 0.01), and significantly positively correlated with QUICK (plasma coenzyme Q10, *β* = 0.04, *p* = 0.08; WBC coenzyme Q10, *β* = 0.08, *p* < 0.01).

## Discussion

Physical exercise may affect glucose dynamics [22] by improving insulin sensitivity [23]. In this study, the athletes showed a higher QUICKI value than the subjects without regular exercise training; however, it is worth noting that athletes had a significantly higher HbA1c level, although the values were within the normal range (Table 2). Lippi et al. [24] also found similar results; elite athletes and cyclists had a significantly lower fasting glucose level but exhibited a trend toward higher HbA1c values than sedentary controls. Recently, Lippi et al. [25] further investigated the fasting glucose and HbA1c values in endurance athletes and found that HbA1c values seemed to be slightly increased (*p* = 0.09) up to 24 h after a 21.1 km marathon run, but the level of fasting glucose was significantly decreased after the run (before run: 4.7 mmol/L decreased to 4.3 mmol/L, *p* < 0.01). It is recommended that athletes consume high glycemic index foods prior to, during and after exercise to elevate the blood glucose levels [4, 26]. Consuming high glycemic index foods can optimize exercise performance and induce adequate muscle glycogen re-synthesis for recovery [4]. In the present study, 28% of the athletes had a high HbA1c level (≥ 5.5%); however, none of the healthy subject had a high HbA1c level. Thus, we suggest that athletes may face the challenge of glycemic control during exercise training and workload, and monitoring their blood glucose levels, such as HbA1c, should be considered. Further studies should detect the susceptibility to diabetes in athletes who consume high glycemic index foods for a long period.

Previous studies have observed that athletes may suffer from coenzyme Q10 deficiency due to sustained heavy physical exertion [27, 28]. Athletes may exhibit a lower coenzyme Q10 status because they have high metabolic requirements; coenzyme Q10 may be depleted during exercise as a result of energy metabolism and limit athletic performance [27, 28]. Studies have further noted that athletes with a higher plasma coenzyme Q10 level (> 2.5 μM) showed better physical performance [29]. In our study, although the level of plasma coenzyme Q10 did not significantly differ from that of healthy individuals, we found that the median level of plasma coenzyme Q10 only reached 0.54 μM in the athletes. Moreover, the WBC coenzyme Q10 level was significantly lower in the athletes than in the healthy individuals (Fig. 1). WBC with nuclei may possess coenzyme Q10 in mitochondria; therefore, it seems that the coenzyme Q10 status was low in the mitochondria in these athletes. A high coenzyme Q10 status may enhance the peak power production of athletes [27, 29]. In this study, athletes without supplementation showed a low marginal coenzyme Q10 status, and adequate supplementation to improve their coenzyme Q10 status should be assessed.

Coenzyme Q10 is a crucial lipid-soluble antioxidant that can regulate glycemia through its antioxidant capacity to inhibit nuclear factor-κB (NF-kB) expression, reduce inflammation, and improve insulin sensitivity [5, 11, 30]. In the present study, we found significant correlations between coenzyme Q10 status and glucose parameters (Fig. 2) and antioxidant capacity in athletes (Table 3). We examined the correlations between antioxidant capacity and glucose parameters in athletes (data not shown). The antioxidative enzymes (CAT and GPx) activity and serum TAC were significantly negatively correlated with fasting glucose (*p* < 0.01), HbA1c (*p* < 0.01), and HOMA-IR (CAT, *p* = 0.01; serum TAC, *p* < 0.01) and positively correlated with QUICKI (CAT, *p* = 0.01; serum TAC, *r* = 0.53, *p* < 0.01). As a result, an adequate coenzyme Q10 status could provide better antioxidant capacity and glycemic control in athletes.

## Conclusions

This study is the first to investigate the relationship between coenzyme Q10 status and blood glucose and antioxidative capacity in athletes. Although we could not determine a causal effect in this cross-sectional study, we found that athletes had a marginal coenzyme Q10 deficiency and that the level of WBC coenzyme Q10 may be associated with glycemic control and antioxidant capacity. Further interventional sport nutrition studies are needed to determine the adequate dose of coenzyme Q10 supplementation in athletes to optimize their coenzyme Q10 status to improve athletic performance and recovery during exercise.

## Data Availability

The datasets generated and/or analyzed during the current study are available from the corresponding author on reasonable request.
